# Patent Foramen Ovale-Induced Platypnea-Orthodeoxia Syndrome: A Case Report and Literature Review

**DOI:** 10.7759/cureus.32203

**Published:** 2022-12-05

**Authors:** Faisal F Alotaibi, Rakan M Alotaibi, Mohammed E Almalki, Mansour D Alhassani, Fahad S Almuqati, Raad A Aldahhas, Wafa H Alameer, Waleed A Hafiz

**Affiliations:** 1 Department of Medicine, Al Noor Specialist Hospital, Makkah, SAU; 2 Department of Medicine, College of Medicine, Umm Al-Qura University, Makkah, SAU; 3 Department of Medicine, Yanbu General Hospital, Yanbu, SAU

**Keywords:** hypoxia, transcatheter repair, transcatheter closure device, internal medicine, adult congenital heart disease, cardiology, structural interventional cardiology, interventional cardiology, patent foramen ovale, platypnea-orthodeoxia syndrome

## Abstract

Platypnea-orthodeoxia syndrome (POS) is a rare condition characterized by dyspnea and hypoxia worsening in the upright position and relieved in the recumbent position. POS can result from anatomical or functional conditions that cause interatrial communication or deformity of the atrial septum. Investigations with imaging and cardiac catheterization can aid in the evaluation. In cases where POS is caused by intracardiac shunting without pulmonary hypertension, closure of the intracardiac shunt can be curative. We report a case of POS in a 54-year-old male who was treated successfully with percutaneous closure of a patent foramen ovale (PFO).

## Introduction

Platypnea-orthodeoxia syndrome (POS) is a rare condition involving positional dyspnea (platypnea) and arterial desaturation (orthodeoxia). Orthodeoxia refers to a situation where arterial oxygen tension drops in the upright position by more than 5% or 4 mmHg [1]. POS is characterized by breathlessness that is alleviated when lying down and aggravated when sitting or standing [2]. The pathophysiology and etiologies of orthodeoxia and platypnea are still not fully understood, but they seem to involve both a functional and an anatomical component [3]. In general, causes can be divided into extracardiac and intracardiac shunting. One or more of the following conditions can cause intracardiac shunting: patent foramen ovale (PFO), atrial septal defect (ASD), atrial septal aneurysm with septal fenestration, partial anomalous pulmonary venous connection, transposition of the great vessels, and unroofed coronary sinus [1]. The most common cause of intracardiac shunting is a PFO [4]. Ventilation-perfusion mismatch, pulmonary shunting, or a combination of these may all be considered extracardiac shunting [2]. The most frequent anatomical cause of an interatrial shunt is PFO, which is commonly asymptomatic; however, patients might present with paradoxical embolism resulting in a stroke, myocardial infarction, or visceral or peripheral ischemia [5]. This report presents a case of POS due to PFO without pulmonary hypertension that underwent percutaneous intervention.

## Case presentation

A 54-year-old male with known chronic liver disease secondary to hepatitis B since the age of 13, who had an ischemic stroke five years ago, with no residuals, presented to the emergency room complaining of subjective fever, cough, and shortness of breath associated with platypnea for one day. The cough was nonproductive, and no aggravating or relieving factors were noted. The patient had no history of chest pain, nausea, vomiting, heartburn, jaundice, hematemesis, headache, or visual changes. A review of other systems was unremarkable. He had no history of contact with sick patients and was not known to have any allergies. His past medical history revealed one episode of diarrhea that had been black in color and had resolved spontaneously. He had no history of surgeries and no history of passive or active smoking.

On initial physical examination, the patient was oriented, conscious, and alert. His vital signs were as follows - temperature: 36.9 °C; heart rate: 69 beats per minute; blood pressure: 113/70 mmHg; respiratory rate: 32 breaths per minute; oxygen saturation, sitting: 85% on room air, lying down: 95% on room air. The patient had finger clubbing and central cyanosis. No palmar erythema, gynecomastia, distended veins, lower limb edema, or signs of deep vein thrombosis were noted. Abdominal examination showed no tenderness, organomegaly, or stigmata of liver cirrhosis. Chest examination was normal with equal bilateral air entry. Heart auscultation revealed normal S1 and S2 and no added sounds or murmurs. The neurological exam was unremarkable. The electrocardiogram showed normal sinus rhythm. Laboratory studies and serology are presented in Table 1.

**Table 1:** Laboratory and serology test results N/A: not applicable; ALP: alkaline phosphatase; ALT: alanine aminotransferase; AST: aspartate aminotransferase; Anti-HBs: hepatitis B surface antibody; HBcAg: hepatitis B core antigen; HBsAg: hepatitis B surface antigen; Anti-IgG HBc: anti-hepatitis B core IgG antibodies; Anti-IgM HBc: anti-hepatitis B core IgM antibodies; HIV antigen/antibody: human immunodeficiency virus antigen/antibody

Test results		
Chemistry	Result	Reference range
White blood count	13.76 x 10³/µL	4–11
Hemoglobin	12.5 g/dL	13–17
Platelet count	155,000 x 10⁹/L	150–400
Sodium	132.7 mmol/L	137–145
Potassium	4.47 mmol/L	3.5–5.1
Blood urea nitrogen	4.78 mmol/L	3.2–7.1
Creatinine	92.5 µmol/L	58–110
Random glucose	96 mg/dL	70–100
Prothrombin time	12.3 seconds	10–14
Partial thromboplastin time	38.9 seconds	23–39
International normalized ratio	0.94	0.85–1.1
Total bilirubin	31.8 µmol/L	3–22
Conjugated bilirubin	12.8 µmol/L	0–7
ALP	87 IU/L	38–126
ALT	33 IU/L	0–50
AST	76 IU/L	15–46
Albumin	23.3 g/L	35–50
Total protein	63.23 g/L	63–82
Serology	Result	Reference range
Anti-HBs	9 mlU/ml	8–12
HBcAg	Reactive	N/A
HBsAg	Reactive	N/A
Anti-IgG HBc	Reactive	N/A
Anti-IgM HBc	Non-reactive	N/A
Hepatitis C virus antibodies	Non-reactive	N/A
HIV antigen/antibody	Negative	N/A

**Figure 1:**
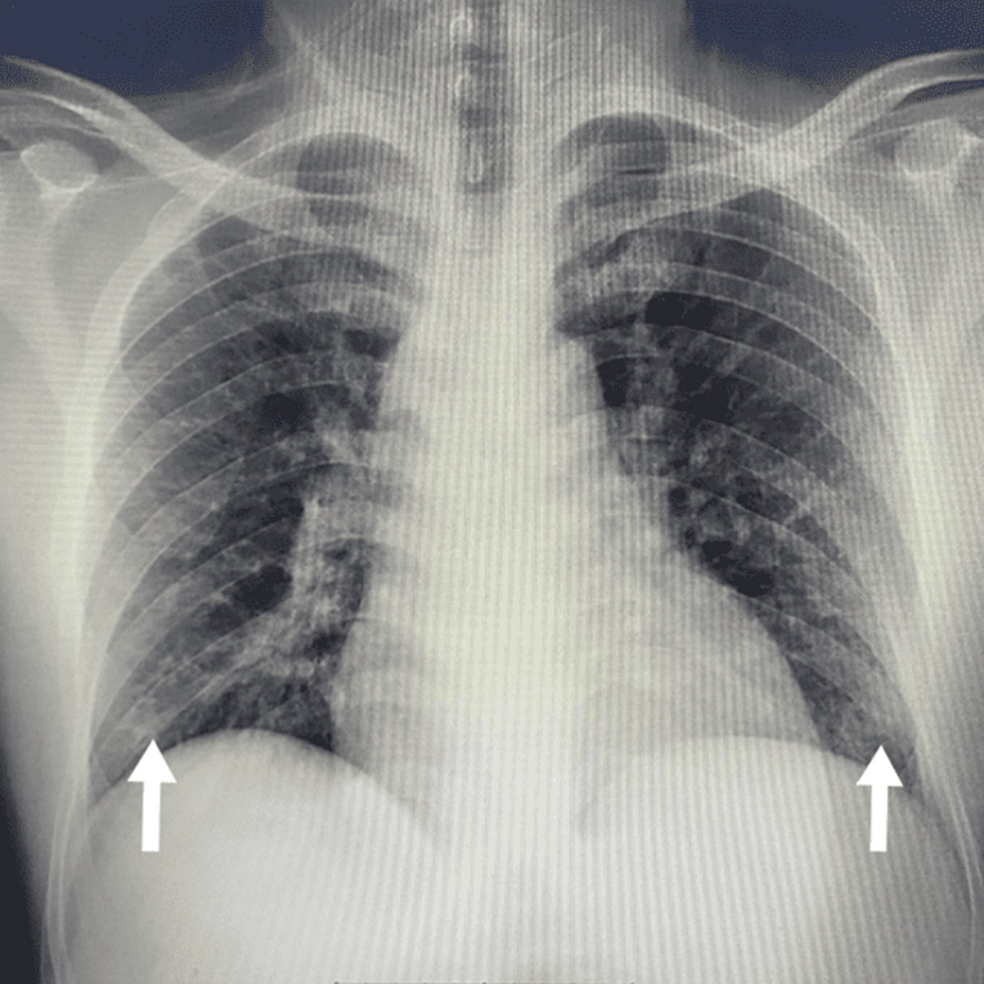
Chest X-ray showing bilateral infiltrations (white arrows)

Chest X-ray showed bilateral infiltrates (Figure 1). The abdominal ultrasound (US) was unremarkable. A thoracic CT scan with contrast was negative for pulmonary embolism. High-resolution CT revealed a small patch of consolidation in the right upper lobe with sub-segmental linear atelectasis as well as patchy consolidation in the left lower lobe. The septic screen revealed a sputum culture positive for *Pseudomonas aeruginosa* sensitive to piperacillin/tazobactam; urine and blood cultures were negative. EGD and colonoscopy were negative. Coronavirus disease 2019 (COVID-19) PCR was also negative.

A transthoracic echocardiogram revealed a normal left ventricular size and normal systolic and diastolic function with an ejection fraction of 55% visually with normal wall motion. Right ventricular size and systolic function were normal. No valvular disease was noted. There was no evidence of aortic disease. Additionally, transthoracic echocardiography with color Doppler showed shunting from right to left atria likely due to PFO or ASD (Video 1). To identify the cause, transesophageal echocardiography with color Doppler and a bubble study using agitated saline was carried out, which showed shunting of saline from the right atrium after two cycles, indicating a substantial right-to-left shunting due to a PFO without pulmonary hypertension (Video 2).

**Video 1:** Doppler transthoracic echocardiography showing right-to-left shunting

**Video 2:** Doppler transesophageal echocardiography with agitated saline study demonstrating a patent foramen ovale with right-to-left shunting

The patient was admitted to the medical ward for treatment of his community-acquired pneumonia and initially received empirical antibiotics therapy comprising intravenous (IV) ceftriaxone 2 g once daily and doxycycline 200 mg once orally. Later, culture-guided antibiotic therapy with piperacillin/tazobactam 4.5 g IV Q6H was carried out for five days. The additional management plan included propranolol 20 mg orally three times per day, spironolactone 100 mg orally once daily, omeprazole 40 mg IV twice daily, and paracetamol 1 G IV when needed for subjective fever. The patient was on 5 L of oxygen by nasal cannula for maintaining normal oxygen saturations of 98% in supine and 96% in upright positions. He was discharged and referred to a specialized center to undergo percutaneous closure of the PFO. Our patient had the clinical features of platypnea and orthodeoxia with oxygen saturation of 85% at an upright position due to PFO. Therefore, a percutaneous closure procedure was performed. He had a 25-mm AMPLATZER™ PFO-occluder device placed under intracardiac echocardiographic guidance, and there were no intraprocedural complications while implanting the device. The patient had no residual shunting following the closure, and his POS symptoms significantly diminished. Upon discharge, he was instructed to see his cardiologist within two weeks. The patient was compliant with follow-up as his blood oxygen saturation was not affected by different body positions. His oxygen saturation improved to 97% in the upright position and to 98% when supine as measured by a pulse oximeter.

## Discussion

POS is a rare disorder that can result from the following four pathological origins: intracardiac shunting, intrapulmonary shunting, ventilation-perfusion mismatch, or a combination of the aforementioned [1,2]. The prevalence of POS due to PFO or ASD is currently unknown [3], and the precise pathophysiological mechanism behind POS remains uncertain. However, various theories have been suggested, most of which agree that there is a correlation between pre-existing interatrial communication and anatomical similarities related to mechanical factors. Most cases of cardiac POS result from the coexistence of an interatrial septal communication (such as ASD, PFO, and fenestrated interatrial septum) associated with a functional and/or structural defect in the chest or abdomen. In POS, right-to-left cardiac shunting occurs mostly with normal right-sided cardiac pressures. Normally in adults, the pressure in the left atrium is slightly greater than that in the right atrium. Therefore, despite the presence of a PFO or an ASD, there is minimal or no right-to-left shunting. Conversely, when the right atrial pressure becomes greater than the left atrial pressure, right-to-left shunting may take place through an interatrial septal defect. This shunting might occur in acute settings as in pulmonary thromboembolism, right ventricular myocardial infarction, pneumothorax, hydrothorax, pericardial effusion, or post pneumonectomy or in chronic settings such as pulmonary hypertension, severe tricuspid regurgitation, pulmonic valve stenosis [4], or hepato-pulmonary syndrome [6]. Interestingly, a left-to-right shunt that is reversed in response to elevated right atrial pressure or decreased right ventricular compliance can lead to this condition [5,7], as in tortuous aortic root and ascending aorta, and hemidiaphragmatic paralysis [8].

The diagnosis of POS is initially established clinically. A drop in oxygen saturation of over 5% observed in an upright position that improves upon recumbency is suggestive of POS. The next step is to identify the mechanism causing desaturation. Because intracardiac shunting is the most common cause of POS, an echocardiogram with bubble contrast with intravenous agitated saline should be the initial diagnostic test, performed in both recumbent and upright positions. This test allows for the differentiation between intracardiac and extracardiac shunting. An intracardiac shunt is suggested by the presence of bubbles in the left atrium within three cardiac cycles [9]. Conversely, delayed microbubble opacification of the left atrium after three to six cardiac cycles indicates an extracardiac shunt, which most frequently occurs in the pulmonary vasculature [10].

If a transthoracic echocardiogram shows indefinite results, a transesophageal echocardiogram can be performed for direct visualization of the cardiac defect [7]. In highly suspected cases with uncertain echocardiographic imaging, cardiac MRI can be used to detect distorted cardiac anatomy [11]. In the absence of intracardiac findings, intrapulmonary causes should be sought through ventilation-perfusion scanning [12]. If an extracardiac shunt is suggested by an echocardiogram, a chest CT angiography can be done to identify pulmonary arteriovenous malformations (AVM). Pulmonary arteriography is considered the gold standard test to diagnose intrapulmonary shunt causes when other tests are inconclusive [13]. The definitive therapy for intracardiac shunting is the repair of the cardiac anomaly, which may include surgical repair or percutaneous closure of an ASD or a PFO [14]. Treatment for extracardiac shunting focuses on the underlying defect where possible. The most common extracardiac cause of POS is pulmonary AVM. Patients with symptomatic pulmonary AVM with a feeding artery larger than 2-3 mm are treated mostly by pulmonary artery embolization [15]. We believe POS is an underdiagnosed condition due to the nature of its clinical presentation, and a high index of suspicion is recommended in patients with unexplained hypoxia and shortness of breath. Table 2 summarizes the key diagnostic and management points for similar reported cases of POS.

**Table 2:** Review of our case and three similar POS cases POS: platypnea-orthodeoxia syndrome; PFO: patent foramen ovale; ASD: atrial septal defect; TEE: transesophageal echocardiogram; CT: computed tomography

Case	Clinical presentation	Investigation (diagnostic)	Type of shunting	Management
Case 1 [16]	A 63-year-old male presented complaining of progressive dyspnea over six weeks. The patient felt the least dyspneic in the supine position	TEE revealed PFO	Right-to-left intracardiac shunt	The patient developed pulseless electrical activity secondary to extensive tumor emboli occluding numerous pulmonary arteries in both lungs before a planned PFO closure
Case 2 [17]	A 79-year-old female presented with progressive exertional dyspnea of two months' duration	TEE revealed ostium secundum type ASD	Left-to-right intracardiac shunt	The patient underwent percutaneous transcatheter closure of the defect with an AMPLATZER™ device
Case 3 [18]	A 76-year-old female presented to the emergency department with a fever, cough, and dyspnea	Chest CT scan revealed interstitial pneumonia. TEE was negative for an intracardiac or intrapulmonary shunt	Fibrotic evolution of interstitial pneumonia extracardiac shunt	The patient was treated for four weeks with high doses of steroids
Our case	A 54-year-old male presented to the emergency room complaining of subjective fever, cough, and shortness of breath associated with platypnea for one day	TEE revealed PFO	Right-to-left intracardiac shunt	A percutaneous closure procedure was carried out

## Conclusions

We discussed a case of PFO-induced POS. We believe that POS is an underrecognized condition with unknown prevalence. Therefore, it is important to suspect this syndrome in patients presenting with unexplained hypoxia and to proceed with appropriate investigations. We recommend further research focusing on implementing diagnostic criteria to increase the likelihood of early diagnosis and estimate the condition's prevalence.
